# Metabolites and Free Fatty Acids in Japanese Black Beef During Wet Aging

**DOI:** 10.3390/metabo15020094

**Published:** 2025-02-03

**Authors:** Shuji Ueda, Yuka Yoshida, Yuka Tateoka, Biniam Kebede, Masakazu Shinohara, Hiroki Nakanishi, Itsuko Fukuda, Yasuhito Shirai

**Affiliations:** 1 Department of Agrobioscience, Graduate School of Agricultural Science, Kobe University, Kobe 657-8501, Japanitsuko@silver.kobe-u.ac.jp (I.F.); shirai@kobe-u.ac.jp (Y.S.); 2 Japan Meat Science and Technology Institute, Inc., Tokyo 150-0013, Japan; 3 Department of Food Science, University of Guelph, Guelph, ON N1G 2W1, Canada; biniam@uoguelph.ca; 4 Department of Food Science, University of Otago, P.O. Box 56, Dunedin 9054, New Zealand; 5 The Integrated Center for Mass Spectrometry, Kobe University, Kobe 650-0017, Japan; mashino@med.kobe-u.ac.jp; 6 Lipidome Lab Co., Ltd., Akita 010-0825, Japan; hnakani@lipidome.jp

**Keywords:** Japanese Black, wet aging, phospholipids, flavor, beef, free fatty acids, lipase, PLA, kokumi

## Abstract

**Background:** Japanese Black beef is known for its high intramuscular fat content, an important factor in its distinctive Wagyu aroma. Wet aging, which involves vacuum-packing meat and storing it at low temperatures, enhances flavor, texture, and tenderness and is essential for maintaining and improving meat quality. In this study, changes in metabolites and lipid profiles were investigated during the wet aging of Japanese Black and Holstein beef. **Methods/Results:** Gas chromatography–mass spectrometry identified 113 metabolites in Japanese Black beef and 94 in Holstein beef, with significant increases in metabolites like aspartic acid and maleic acid over the aging period. Regarding lipid composition, total free fatty acids significantly increased with wet aging, with Japanese Black beef showing significantly higher concentrations of oleic and linoleic acids than Holstein beef. Additionally, lipid analysis by liquid chromatography–mass spectrometry revealed a reduction in specific phospholipids, particularly lysophosphatidylcholine (LPC) and lysophosphatidylethanolamine (LPE), with notable decreases in LPC (18:1), LPC (18:2), LPE (18:1), and LPE (18:2). **Conclusions:** These results suggest that wet aging influences the stability of membrane lipids, facilitating the degradation of phospholipids into free fatty acids, and improving the flavor of Japanese Black beef.

## 1. Introduction

Japanese Wagyu, specifically Japanese Black cattle, is a highly regarded breed in Japan [1,2]. Japanese Black cattle are the offspring of bulls selected through meat quality evaluation tests. Japanese Black cattle are characterized by the high accumulation of intramuscular fat (marbling) in skeletal muscles, distinguishing them from other breeds. As intramuscular fat increases, the sweetness of the beef is more distinctly perceived in sensory evaluations [3]. Additionally, intramuscular fat generates sweet flavors, including lactones, which contribute to a distinct sweet flavor (commonly called the Wagyu aroma) [4].

Holstein-Friesian (Holstein) cattle are one of the most common dairy cattle breeds. Despite their primary use for milk production, Holstein steers are also fattened earlier (average age 20 months) than Japanese Black cattle (28 months or older) and utilized to produce low-cost beef. Holstein steers generally produce leaner meat and contain less intramuscular fat than Japanese Black cattle [5]. In a sensory evaluation comparing Japanese Black and Holstein beef, Japanese Black beef exhibited a stronger sweetness than Holstein beef [6]. Additionally, odor analysis revealed that lactones were detected at significantly higher levels in Japanese Black beef than in Holstein beef [4].

This difference in the amount of intramuscular fat is attributed to the genetic characteristics of the breed [7]. Physiological differences in the endocrine system [1] and variations in microRNA [8] production are believed to play an important role in the formation of intramuscular fat. Previous research using gas chromatography–mass spectrometry (GC/MS) to analyze metabolites has shown that Japanese Black beef contains higher levels of several metabolites (e.g., medium-chain fatty acids) compared to Holstein beef [9,10]. Therefore, the Holstein breed, which exhibits different meat quality characteristics, is often used as a comparison in research on the Japanese Black cattle.

After slaughter, beef is processed into cuts, typically requiring 2–4 weeks to reach consumers through various distribution channels in the Japanese market. Refrigerating beef after slaughter enhances sensory characteristics, such as taste, flavor, and tenderness [11]. This process, known as postmortem aging, is a key factor in meat quality. There are two aging methods: wet and dry aging [12]. Dry aging is conducted in a refrigerator under low-humidity conditions, providing a unique aged flavor to the meat [13]. In contrast, wet aging involves vacuum-packing meat in an oxygen barrier film and refrigerating it. In standard meat distribution, beef is commonly packaged in plastic film and refrigerated for transport, effectively acting as wet aging [14]. During aging, proteins, carbohydrates, and nucleic acids undergo endogenous enzymatic degradation [15]. This enzymatic breakdown increases the concentration of free amino acids, while the degradation of nucleic acids produces inosinic acid, a nucleotide-related metabolite. These metabolites contribute to the umami taste, enhancing the overall quality of the meat. [16]. Postmortem aging is applied to all livestock species, although aging rates vary depending on breed, meat cut, and storage temperature [17]. The optimal wet aging conditions for each breed are an important issue for the meat industry.

Recent advances in metabolomics analysis have facilitated detailed research into the nutritional components of meat in various livestock species. Metabolomics allows for the comprehensive analysis of metabolites and has significantly deepened our understanding of postmortem aging processes [18]. In previous research, we used crossbred Wagyu (Japanese Black × Holstein) to investigate how metabolite changes due to wet aging affect the taste of beef [19]. During this process, we found that, in addition to the known free amino acids and nucleotide-related metabolites, the amount of free fatty acids (FFAs) also increased significantly during wet aging [19]. FFAs are recognized for influencing the quality of processed meat products such as cured meats [20] and fermented sausages [21]. However, their role and formation mechanisms in fresh meat remain poorly understood.

In this study, we conducted metabolomics analysis on purebred Japanese Black and Holstein beef using GC/MS and liquid chromatography–mass spectrometry (LC/MS) to compare their nutritional components during wet aging, focusing on FFAs and membrane phospholipids, and to clarify the effect of wet aging on meat quality in Japanese Black beef.

## 2. Materials and Methods

### 2.1. Beef Samples and Wet Aging Conditions

Japanese Black and Holstein beef were purchased commercially from selected farmers through meat producers. Five Japanese Black cattle were 28-month-old steers (origin: Nagasaki Prefecture, Japan) with a marbling score of 4 (according to the Japan Meat Grading Association standard). Five Holstein cattle were 20-month-old steers (Aomori Prefecture, Japan). The concentrate feed for fattening each breed consisted of a general composition commonly used by Japanese livestock farmers, including corn, rice bran, and soybean meal.

Both sides of the striploin (Loin; longissimus thoracis) and top round (Round; adductor muscle) were collected from each carcass. Sample handling followed previously reported procedures [19], with the right side of each carcass used for metabolomic analysis and the left for other component analyses (Figure S1). Each Loin and Round block was divided into five portions (weighing approximately 2 kg each) and vacuum-packed in an oxygen-barrier film (Varialon S, Asahi Kasei Corporation, Tokyo, Japan). Wet aging conditions were maintained in a refrigerated set at 0 °C and 75% humidity for 0, 10, 20, 30, and 40 days at a meat processing center (Ogawa Chikusan Co., Ltd., Tokyo, Japan). Wet-aged samples were rapidly frozen and stored until the experimental date.

### 2.2. Metabolomics Analysis

A sample was taken from the interior of the frozen beef and then finely shredded, and crushed using a multi-bead shocker (μT-48, Tokken Inc., Chiba, Japan). For each 10 mg of crushed beef, 1 mL of a solvent mixture (methanol: chloroform: water, 2.5: 1: 1) containing 5 mg of sinapic acid (Fujifilm Wako Pure Chemicals Co., Osaka, Japan) as internal standard was added. The mixture was stirred at 1500 rpm for 5 min on a rotator. After centrifuging at 15,000× *g* for 5 min, the supernatant was completely dried using a centrifugal evaporator and freeze-dried for 16 h. The dried samples were dissolved in 80 μL of pyridine containing 20 mg/mL methoxyamine hydrochloride (Sigma-Aldrich Japan G.K., Tokyo, Japan) with sonication, shaken at 1200 rpm for 30 min at 30 °C, and derivatized with N-methyl-N-trimethylsilyltrifluoroacetamide (GL Sciences, Tokyo, Japan) at 37 °C for 30 min. After centrifugation at 16,000× *g* for 5 min at 4 °C, 50 μL of the supernatant was subjected to GC/MS analysis.

GC/MS analysis was conducted using a GCMS–QP2010 Ultra system (Shimadzu Corporation, Kyoto, Japan) equipped with a DB-5 capillary column (30 m × 0.25 mm, 1.0 μm film thickness; Agilent Technologies, Santa Clara, CA, USA). The column temperature was held at 100 °C for 4 min and then raised by 10 °C/min to 320 °C and held at 320 °C for 6–11 min. The front inlet temperature was held at 280 °C. The helium gas flow rate was 39.0 cm/s. The injected sample volume was 1 μL. The interface and ion-source temperatures were 280 °C and 200 °C, respectively. Mass spectra were analyzed using the GC/MS Metabolite Database v.2 (Shimadzu). The amount of each metabolite was calculated based on the intensity ratio (peak area of metabolites/peak area of sinapic acid) indicated in the GC/MS spectrum [19].

### 2.3. Analysis of Total Fatty Acids and Free Fatty Acids

Frozen beef samples (5 g) were finely ground and treated with an organic solvent mixture (methanol: chloroform, 1:1) following the Folch method [22] to extract the lipid fraction. Dihomo-γ-linolenic acid (Fujifilm Wako Pure Chemical, Osaka, Japan) was used as an internal standard. FFAs were isolated using a solid-phase extraction column (InertSep SI, GL Sciences Co., Tokyo, Japan) and nitrogen-dried. The lipid fraction was further processed for FFAs using a solid-phase extraction column (Bond Elut NH2; Agilent Technologies, Santa Clara, CA, USA). FFA extraction involved conditioning the column with hexane, washing it with chloroform: isopropanol (2:1), and eluting the fatty acids with 2% acetic acid in diethyl ether. The eluted fatty acids were dried under nitrogen.

Both total fatty acids and FFAs were methyl esterified with a boron trifluoride (14%)—methanol solution (Sigma-Aldrich Japan G.K., Tokyo, Japan). Samples were then extracted with saturated sodium chloride in n-hexane, dehydrated with anhydrous sodium sulfate, and analyzed using gas chromatography with a flame ionization detector (GC-2010plus, Shimadzu Corporation, Kyoto, Japan). Separation was performed on an SP-2560 column (0.25 mm ID, 100 m length, 0.20 μm film thickness; Supelco Inc., Bellefonte, PA, USA). The analytical conditions were as follows: inlet temperature 250 °C, detector temperature 250 °C, and column temperature program: 180 °C (150 min), gradient 10 °C/min to 220 °C [19].

### 2.4. Analysis of Phospholipids Molecules

The sample was ground in methanol and suspended at 100 mg/mL. Then, 10 μL of the suspension was mixed with chloroform, methanol, and water for phase separation, according to the Bligh and Dyer method [23]. The mixture was thoroughly vortexed and centrifuged at 3000× *g* for 10 min to separate the organic and aqueous phases. The lower organic phase, containing the total lipids, including phospholipids, was carefully collected and dried under nitrogen gas.

This lipid fraction was reconstituted in methanol and analyzed with LC/MS/MS using a DIONEX Ultimate 3000 system (Thermo Fisher Scientific KK, Tokyo, Japan) coupled to an L-column3 C18 metal-free column (2 mm ID, 100 mm length, 2 μm particle size; Chemicals Evaluation and Research Institute, Tokyo, Japan) and a Q Exactive Plus mass spectrometer (Thermo Fisher Scientific KK, Tokyo, Japan). The high-performance liquid chromatography method used solvent A (5 mM ammonium formate with 0.05% ammonium hydroxide in a solvent mixture [2-propanol = 5:1:4]), and solvent B (5 mM ammonium formate, 0.05% ammonium hydroxide, and 2-propanol). The column was maintained at 40 °C with an injection volume of 10 μL and a flow rate of 0.1 mL/min. MS was conducted in Full MS/dd-MS2 mode, capturing spectra between 200 m/z and 1800 m/z in positive ion mode. For internal standardization, the following parameters were utilized: TG (15:0/18:0/15:0) d5 for TG, PC (18:1d7/15:0) for LPC and PC, PE (18:1d7/15:0) for PE and LPE, DG (17:0/17:0) d5 for DG, PS (18:1d7/15:0) for PS, PI (18:1d7/15:0) for both lysophosphatidylinositol and PI, MG (18:1d7) for MG, PG (18:1d7/15:0) for PG, CL (14:0/14:0/14:0/14:0) for CL, SM (d18:1/12:0) for SM, and Cer (d18:1/12:0) for Cer. All standards were acquired from Avanti Polar Lipids, Inc. (Alabaster, AL, USA). The molecular species of lipids and membrane phospholipids were identified using the lipid analysis software Lipid Search v.4.2.23 (Mitsui Knowledge Industry Co., Ltd., Tokyo, Japan).

### 2.5. Statistical Analyses

Principal component analysis (PCA) was performed using SIMCA 14.1 software (Infocom, Tokyo, Japan). Scaling of the PCA was performed in UV mode. Statistical significance was determined using Student’s *t*-test in Microsoft 365 Excel (Microsoft Japan, Tokyo, Japan) or the one-way ANOVA method in Bellcurve (Social Survey Research Information, Tokyo, Japan).

## 3. Results

### 3.1. Metabolite Changes During Wet Aging

**Figure 1:**
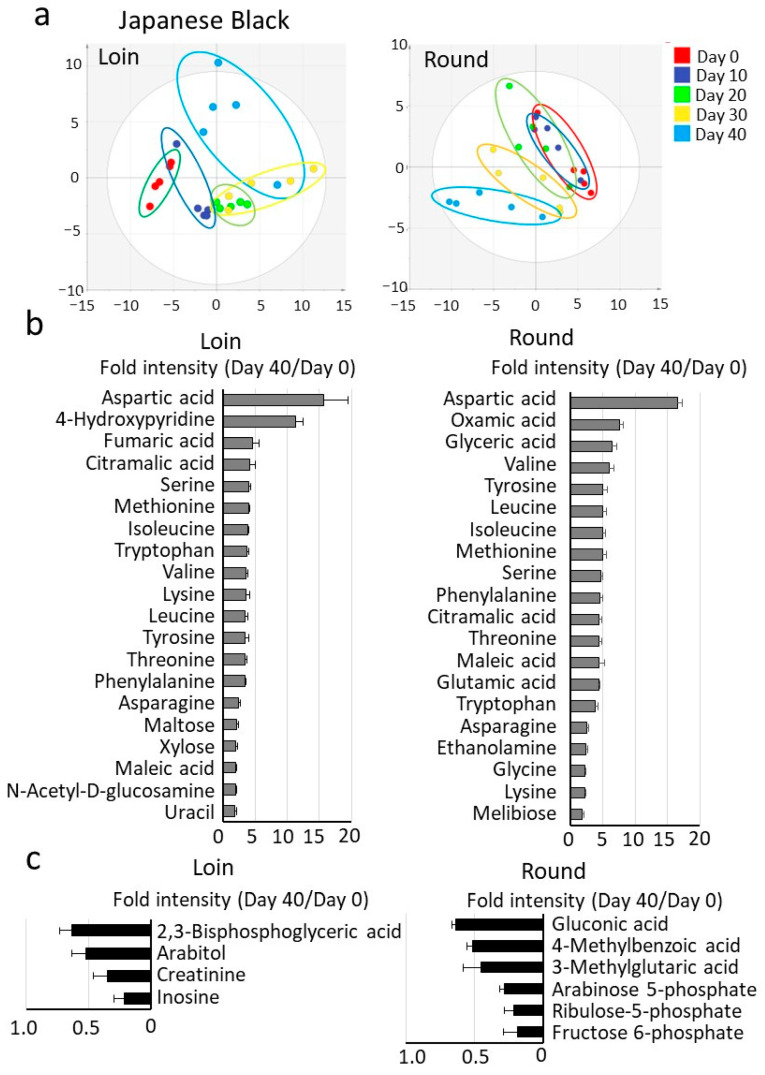
Metabolomics analysis of Japanese Black cattle during wet aging. (**a**) Score plots of principal component analysis of the detected metabolites. Vacuum-packed longissimus thoracis (Loin) and adductor muscle (Round) samples of Japanese Black cattle were refrigerated from Day 0 through Day 40. The water-soluble fraction of the sample was analyzed by gas chromatography–mass spectrometry. The score plot classified the Loin and Round sample groups into five groups according to wet aging time. (**b**) The relative increase in the top 20 metabolites was significantly increased by cold storage. (**c**) Metabolites with significantly decreased relative levels by cold storage. Values are averages of the relative values from Day 0 to Day 40. Error bars represent the mean ± SE. Significant differences were observed for all metabolites in the graph (Student’s *t*-test; *n* = 5; *p* < 0.05).

**Figure 2:**
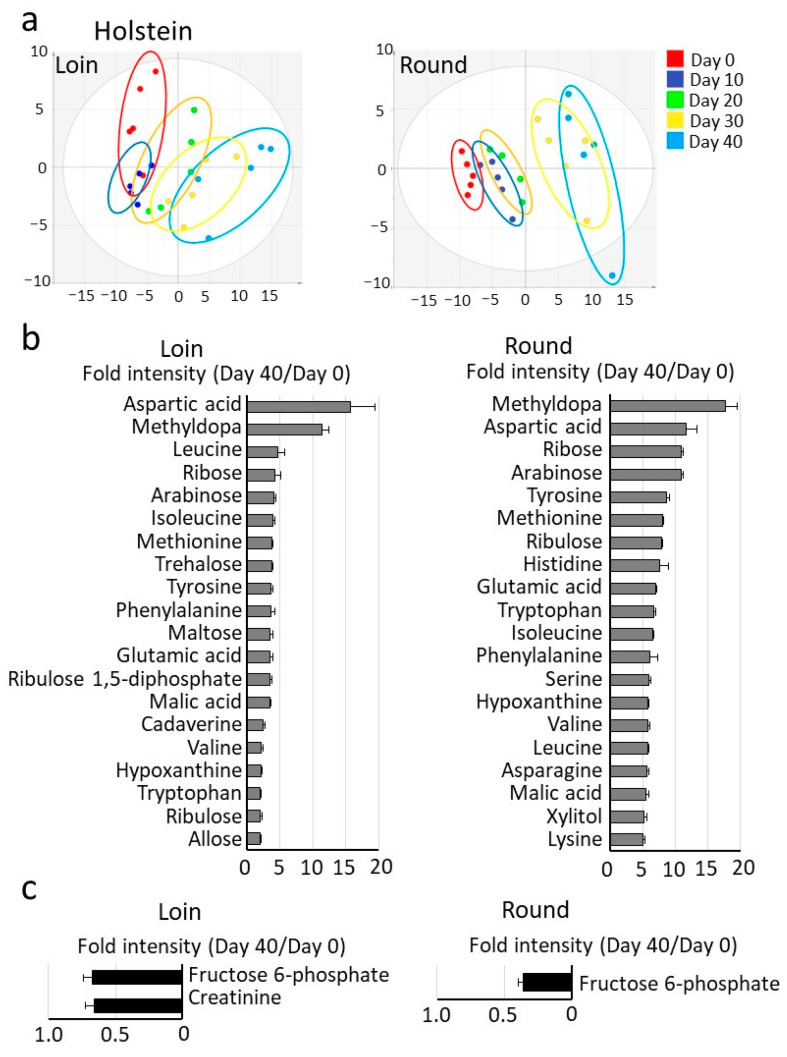
Metabolomics analysis of Holstein cattle during wet aging. (**a**) Score plots of principal component analysis of the detected metabolites. Vacuum-packed longissimus thoracis (Loin) and adductor muscle (Round) samples of Holstein cattle were refrigerated from Day 0 through Day 40. The water-soluble fraction of the sample was analyzed by gas chromatography–mass spectrometry. The score plot classified the Loin and Round sample groups into five groups according to wet aging time. (**b**) The relative increase in the top 20 metabolites was significantly increased by cold storage. (**c**) Metabolites with significantly decreased relative levels by cold storage. Values are averages of the relative values from Day 0 to Day 40. Error bars represent the mean ± SE. Significant differences were observed for all metabolites in the graph (Student’s *t*-test; *n* = 5; *p* < 0.05).

According to Japanese market standards, the safe storage period for vacuum-packed beef stored in a refrigerator is approximately 45 days. In this study, wet aging was carried out from Day 0 (four days post-slaughter) to Day 40 in the refrigerator after cutting a block from the carcass (Figure S1). Metabolomics analysis detected metabolites, including carbohydrates, amino acids, and organic acids in Japanese Black beef (113 metabolites) and Holstein beef (94 metabolites). The metabolite data were subjected to PCA, visually categorizing the correlation between detected metabolites and wet aging days for Japanese Black beef (Figure 1a) and Holstein beef (Figure 2a). The PCA score plot is divided into five groups according to the wet aging period. The average coefficient of variation (CV) for data during the wet aging was 41.3% for Japanese Black Loin and 44.8% for Round, and 30.6% for Holstein Loin and 31.6% for Round. The level of most metabolites in Japanese Black and Holstein beef increased with cold storage time. Figure 1b and Figure 2b show the top 20 metabolites, with significant differences between Day 0 and Day 40. Figure 1c and Figure 2c show decreased metabolites with significant differences between Day 0 and Day 40.

**Figure 3:**
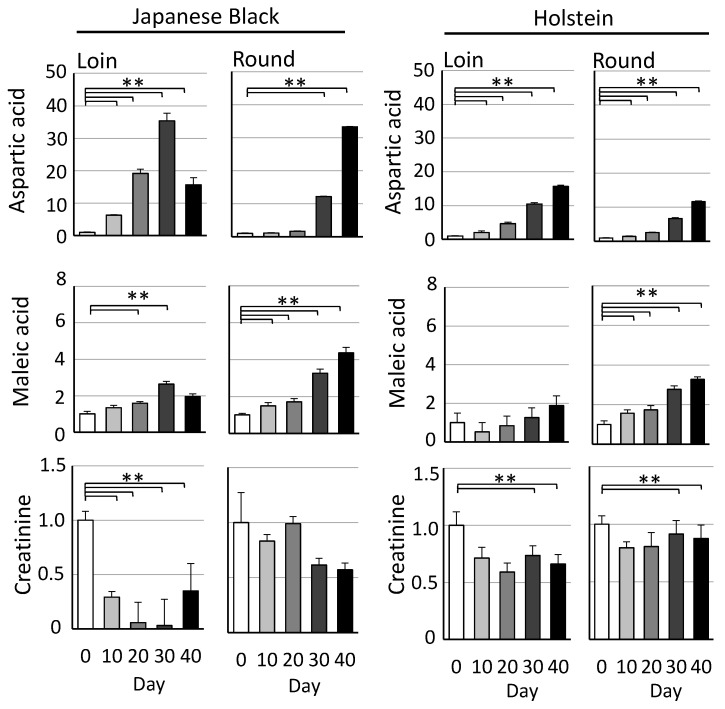
Metabolites change during wet aging. Graphs show representative metabolites detected in Japanese Black and Holstein beef, with values expressed relative to Day 0. Error bars represent the mean ± SE. Significant differences are indicated (Dunnett’s test; *n* = 5; ** *p* < 0.01).

Free amino acids and tricarboxylic acid cycle metabolites were among the metabolites whose levels increased significantly during cold storage. Specifically, amino acids (aspartic acid, leucine, tyrosine, serine, isoleucine, among others) and tricarboxylic acid cycle metabolites (maleic acid, fumaric acid, citric acid) showed a marked increase. In contrast, a significant decrease in metabolites such as creatine and fructose 6-phosphate was observed. In comparing Japanese Black and Holstein beef, Japanese Black beef tended to have higher levels of fructose, glucose, lactic acid, glycerol, and nucleotide-related metabolites. Graphs display representative metabolites that showed significant increases or decreases during wet aging (Figure 3). Other key metabolites in wet aging are shown in the (Supplementary Data Tables S1 and S2).

### 3.2. Free Fatty Acid Changes During Wet Aging

**Figure 4:**
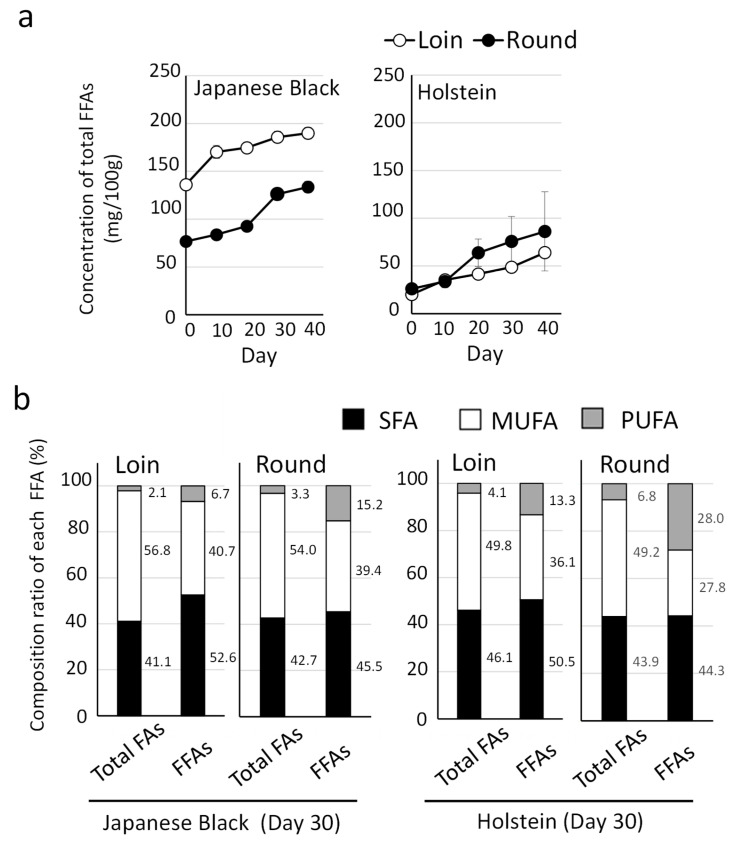
Free fatty acids change over time during wet aging. (**a**) Mean values of free fatty acids (FFAs) analyzed by high-performance liquid chromatography (*n* = 5) are presented. Error bars represent the mean ± SD. (**b**) The percentage composition of polyunsaturated fatty acids (PUFAs), monounsaturated fatty acids (MUFAs), and saturated fatty acids (SFAs) in total fatty acids (Total FAs) and FFAs at 30 days of cold storage is shown.

Next, we quantified FFAs in Japanese Black and Holstein beef. Wet aging resulted in an increasing trend in total FFA levels in Japanese Black and Holstein beef, depending on the cold storage (Figure 4a). A comparison between Japanese Black and Holstein beef revealed that the total FFA levels in Japanese Black beef were significantly higher than those in Holstein beef. In the comparison between Loin and Round, the level of FFA in the Loin was higher than that in the Round in the Japanese Black cattle. However, there was no significant difference in the level of FFA between the Loin and Round in the Holstein cattle.

Next, the fatty acid composition of total FAs and FFAs after 30 days of wet aging is presented (Figure 4b). The proportion of polyunsaturated fatty acids (PUFAs) in the total FFAs was 6.7% in the Loin and 15.2% in the Round of Japanese Black beef. Similarly, the proportion of PUFAs in Holstein beef was 13.3% in the Loin and 28.0% in the Round. In both Japanese Black and Holstein beef, the percentage of PUFAs in FFAs tended to be higher in the Round than in the Loin.

**Figure 5:**
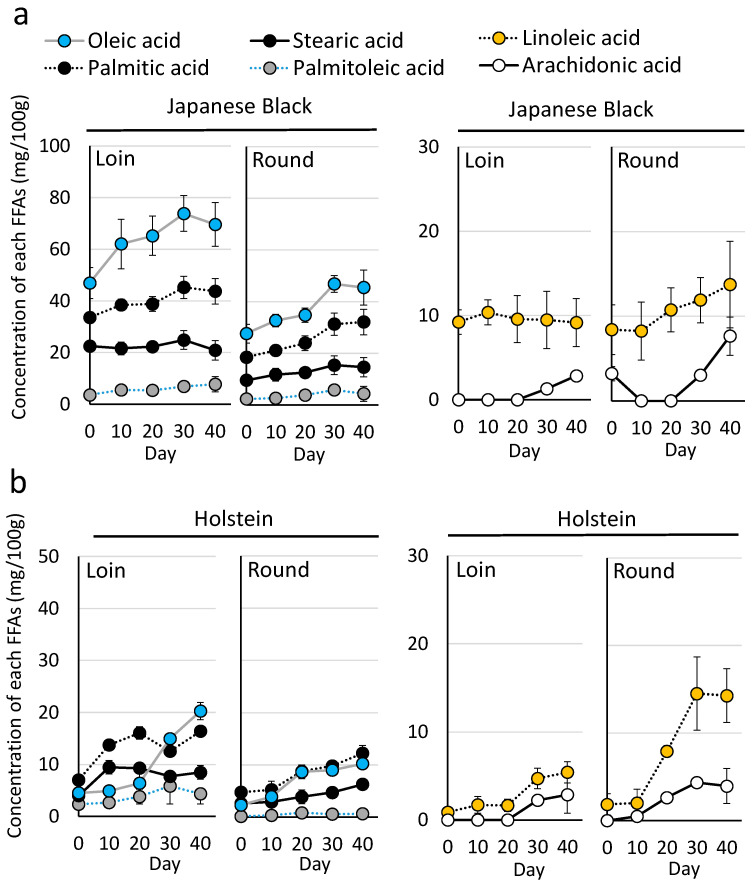
Temporal changes in free fatty acid content during wet aging. (**a**) Free fatty acids detected in Japanese Black beef. (**b**) Free fatty acids detected in Holstein beef. The graph represents the mean values of free fatty acids detected in the cattle (*n* = 5 for each group). Error bars represent the mean ± SD.

The concentration of individual FFAs increased depending on cold storage time (Figure 5a). In Japanese Black beef, oleic acid, palmitic acid, stearic acid, and linoleic acid were the predominant FFAs. The concentration of free oleic acid was Loin (46.9 ± 6.0 mg) and Round (27.6 ± 3.6 mg) at Day 0, and Loin (73.8 ± 7.0 mg) and Round (46.9 ± 3.2 mg) at Day 30 of wet aging. The concentration of free linoleic acid was Loin (9.2 ± 1.5 mg) and Round (8.4 ± 2.9 mg) at Day 0, and Loin (9.5 ± 3.4 mg) and Round (11.9 ± 2.7 mg) on Day 30. The values for each fatty acid are shown in the Table S3.

In Holstein beef, palmitic acid and stearic acid were predominant at the early stages of wet aging, while oleic acid and linoleic acid increased after 30 days (Figure 5b). The free oleic acid was Loin (4.5 ± 0.7 mg) and Round (4.6 ± 2.6 mg) at Day 0, and Loin (15.0 ± 0.7 mg) and Round (18.0 ± 3.2 mg) in Holsteins on Day 30. In contrast, the free linoleic acid was Loin (0.9 ± 0.3 mg) and Round (1.9 ± 1.2 mg) at Day 0, and Loin (4.7 ± 1.2 mg) and Round (14.5 ± 4.2 mg) on Day 30. The values for each fatty acid are shown in the table (Table S4).

### 3.3. Phospholipid Changes Due to Wet Aging

We conducted a comprehensive analysis of lipid molecular species, focusing on phospholipids. In our LC-MS/MS analysis, triacylglycerides, phosphatidylcholines (PCs), lysophosphatidylcholines (LPCs), phosphatidylethanolamines (PEs), and lysophosphatidylethanolamines (LPEs) were detected in the total lipid fraction. LC/MS/MS analysis identified 259 lipid molecular species in the Loin and 234 molecular species in the Round of Japanese Black beef. Among these, the phospholipid species with a coefficient of variation (CV) value of 20% or less included 118 out of 380 in the Loin and 113 out of 390 in the Round, indicating relatively low variability. The lipid classification of these molecular species identified by LC/MS/MS and their heatmap are shown in Figure S2.

**Figure 6:**
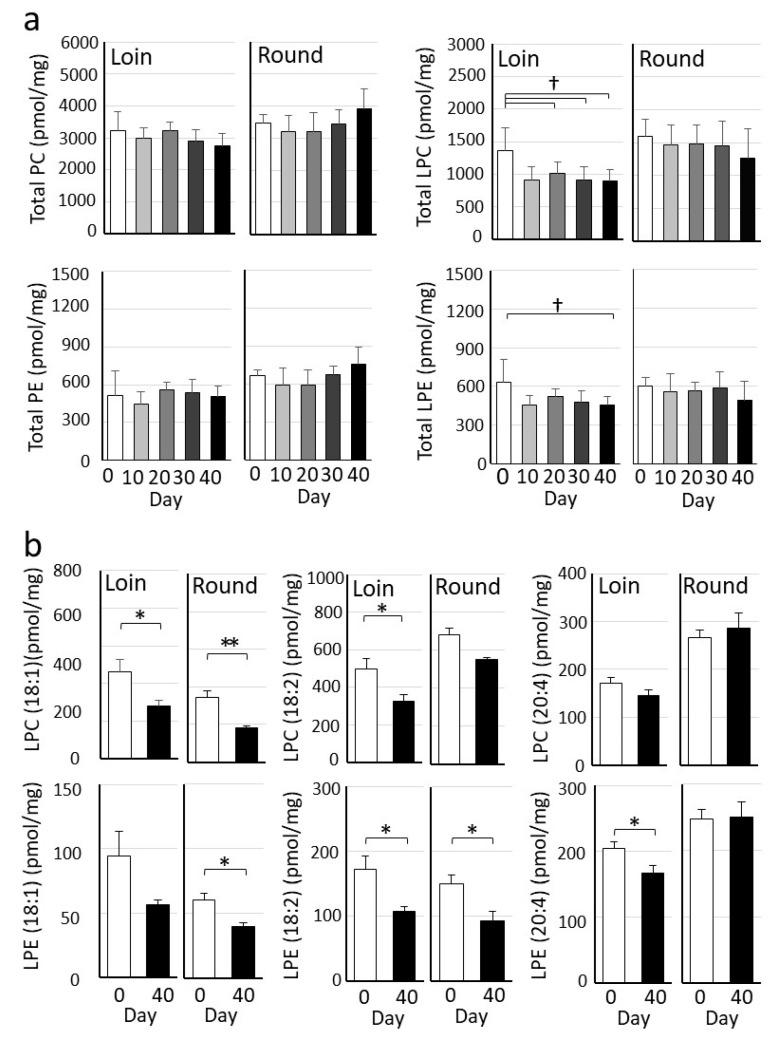
Temporal changes in lipid molecules in Japanese Black beef during wet aging. (**a**) Phospholipids detected in Japanese Black beef. The graphs represent the mean values (pmol/mg) of total phosphatidylcholine (PC), phosphatidylethanolamine (PE), lysophosphatidylcholine (LPC), and lysophosphatidylethanolamine (LPE). The significance of differences was tested using one-way ANOVA (Dunnett’s test; † *p* < 0.05, *n* = 5). Error bars represent the mean ± SE. (**b**) Molecular species of phospholipids with significant differences. The graphs show the mean values (pmol/mg) for LPC and LPE molecular species (Student’s *t*-test; * *p* < 0.05, *n* = 5; ** *p* < 0.01).

Among the detected lipid classes, LPC and LPE, generated by the lipase-mediated products of PC and PE, tended to decrease with extended cold storage (Figure 6a). Furthermore, detailed analyses revealed significant decreases in specific LPC and LPE, including LPC (18:1), LPC (18:2), LPE (18:1), LPE (18:2), and LPE (20:4) (Figure 6b). In the Loin, significant decreases were observed in LPC (18:1), LPC (18:2), LPE (18:2), and LPE (20:4), while in the Round, significant decreases were observed for LPC (18:1), LPE (18:1), and LPE (18:2).

## 4. Discussion

Metabolomics analysis using GC/MS revealed distinct profiles of amino acids, carbohydrates, and organic acids in Japanese Black and Holstein beef. Both breeds showed increased free amino acid content with longer storage periods (Figure 1 and Figure 2). The increase in these metabolites during cold storage was consistent and highly reproducible in other studies [24]. In Japanese Black cattle, the Round contained higher levels of branched-chain amino acids (BCAAs; isoleucine, valine, and leucine) compared to the Loin. In contrast, the Holstein cattle showed high BCAA levels in both Loin and Round. The difference in BCAAs between the Loin and Round of Japanese Black beef is thought to be primarily influenced by the amount of intramuscular fat. BCAAs are bitter-tasting amino acids found in large quantities in the proteins that constitute muscle fibers. BCAAs also contribute to the persistence of the “richness of flavor” of meat [25]. In the previous study [19], wet aging significantly increased the persistence of the meaty richness of flavor of crossbred Wagyu in sensory evaluation tests. The persistence of this flavor, also known as “kokumi”, is a new concept of taste response caused by the binding of peptides to calcium-sensing receptors [26]. The observed increase in BCAA levels during wet aging may be a marker of enhanced richness in the meat flavor.

In a comparison of free amino acids, Japanese Black cattle beef exhibited higher levels than Holstein cattle. This result was further supported by quantitative analysis of free amino acids during wet aging using conventional high-performance liquid chromatography (Figure S3). A possible explanation for this difference is the longer fattening period of Japanese Black cattle (28 months) compared to Holstein cattle (20 months). This extended fattening period is associated with increased maturity of muscle fibers, which has been reported to increase the levels of free amino acids and nucleotide-related metabolites [27].

Furthermore, data indicate that inosinic acid (also known as inosine 5′-monophosphate), a nucleotide-related metabolite associated with umami taste [28], degrades during wet aging, leading to the accumulation of its breakdown products, hypoxanthine via inosine (Figure 1 and Figure 2). These findings were further supported by quantitative analysis using conventional high-performance liquid chromatography (Figure S4). Similar results were also confirmed in the previous studies on crossbred wagyu [19]. The degradation of inosinic acid during wet aging showed no significant differences among Japanese Black, Holstein, and crossbred Wagyu beef. However, in Japanese Black cattle and crossbred Wagyu, the Loin may degrade inosinic acid more rapidly than the Round, with reductions occurring approximately 10 days earlier under our experimental conditions. To illustrate the dynamics of metabolite changes during wet aging, the metabolomics analysis data were visualized using a heatmap (Figure S5). Notably, in Japanese Black beef, the heatmap gradients of metabolite changes in the Loin showed a tendency for earlier peaks than the Round. The wet aging process is influenced by several factors, including refrigerator temperature, the size of the meat block, and the physiological state of the meat [29]. Establishing the optimal wet aging period for Japanese Black beef will require further analysis of additional samples under diverse conditions. Furthermore, during wet aging, residual levels of inosinic acid have been reported to be higher than those observed in dry aging [30]. These results emphasize that the dynamics of umami-related metabolites, such as inosinic acid and free amino acids, are significantly influenced by aging conditions and specific meat cuts [31].

A notable distinction between the breeds was the significant accumulation of FFAs. At the start of wet aging (Day 0), Japanese Black beef contained over 10 times the amount of free oleic and linoleic acids compared to Holstein beef (Figure 5). Wet aging further increased FFA levels in both breeds, with a more pronounced increase in free oleic acid in the Loin and free linoleic acid in the Round. These trends in FFA accumulation were consistent with previous findings in crossbred Wagyu beef [19]. By integrating the results from both studies, it was evident that FFA levels were highest in Japanese Black beef, followed by crossbred Wagyu, and lowest in Holstein beef.

FFAs are more susceptible to oxidation during cooking than triglycerides and phospholipids [32], and they serve as precursors of odor compounds contributing to beef aroma [33]. Furthermore, recent studies have shown that FFAs, such as oleic acid, linoleic acid, and stearic acid, influence taste perception by interacting with specific taste receptors (CD36 and GPR120) located in taste buds [34]. These findings highlight the pivotal role of FFAs in determining the flavor and quality of beef.

The main lipids in beef include triacylglycerides, which form intramuscular fat, and phospholipids that comprise cell membranes, such as the sarcolemma and sarcoplasmic reticulum, [35] and Z disk of muscle fibers [36]. The lipid composition of Japanese Black cattle is characterized by a high proportion of oleic acid, exceeding 50%, compared to approximately 40% in Holstein cattle [33]. Triacylglycerol degradation is mediated by enzymes such as lipoprotein lipase (LPL) and hormone-sensitive lipase (HSL) [37]. These lipases are preferentially expressed in intramuscular fat and subcutaneous fat of Japanese Black beef [38]. The elevated levels of free oleic acid observed in the Loin may be associated with the activity of LPL and HSL in the intramuscular fat present in marbling.

Linoleic acid is unevenly distributed in phospholipids and is degraded from phospholipids by various phospholipase A (PLA) enzymes [39]. There are two families of PLA enzymes, PLA1 and PLA2, which hydrolyze the sn-1 and sn-2 acyl chains of phospholipids such as PC and PE, releasing free fatty acids and lysophospholipids like LPC and LPE. PLA2G16 (also known as adipose-specific PLA2), highly expressed in fat and muscle cells, is well known for degrading phospholipids to release PUFAs. Lipases such as PLA2G16 may contribute to the increase in linoleic acid and arachidonic acid observed during wet aging. Moreover, PLA2G16 has been reported to exhibit genetic polymorphisms, widely observed in breeds such as Nellore cattle [40] and Chinese cattle [41]. Genes encoding lipases could serve as promising marker candidates for enhancing livestock meat quality in the future.

In a previous study on crossbred Wagyu, conventional high-performance liquid chromatography showed no significant decrease in triacylglyceride composition during wet aging [19]. Therefore, we conducted a detailed analysis of lipid molecular species using LC/MS/MS (Figure 6). The total amount of PC, PE, LPC, and LPE decreased during wet aging. While some molecular species showed no significant changes, a notable reduction was observed in lysophospholipids such as LPC (18:1), LPC (18:2), LPE (18:1), LPE (18:2), and LPE (20:4). Lysophospholipids, generated by the cleavage of the sn-2 acyl group by PLA2 [42] and further degraded by lysoPLA1 [43], are associated with FFA release. However, the specific role of lysoPLA1 remains poorly understood, and the lipid metabolic pathways of lysophospholipids in meat are still largely unknown [43].

FFAs are well-known precursors of meat aroma. In meat FFAs undergo lipid oxidation and lipolysis, resulting in the formation of meat-like odor components such as aldehydes, alcohols, and ketones [44]. Furthermore, the cyclization of hydroxy fatty acids generates lactones [45], which contribute to the characteristic sweet aroma unique to Japanese Black beef [46]. The marbling in Japanese Black beef contains an especially high concentration of hydroxy fatty acids [33], considered precursors to lactone production. Based on these findings, it has been hypothesized that during wet aging, unsaturated fatty acids in beef undergo enzymatic oxidation or auto-oxidation, leading to the formation of hydroxy fatty acids [46]. Further research is needed to clarify the link between FFA levels and lactone production during wet aging.

Our metabolomics analysis data showed that Japanese Black beef tends to contain higher levels of carbohydrates such as fructose and glucose than Holstein beef. During the cooking process, in addition to lactones, odor components produced by the Maillard reaction of reducing sugars like fructose and glucose are also significant contributors to beef aroma [47]. It has been reported that Japanese Black beef contains higher glucose levels than other livestock species, contributing to increased odor production [48]. This report is consistent with our metabolomics analysis data and suggests that Japanese Black beef contains higher levels of carbohydrates.

Additionally, using LC-MS/MS, we were the first to demonstrate a decrease in lysophospholipids in Japanese Black beef during wet aging. Regarding the relationship between postmortem aging and phospholipids, it has been reported that LPE (20:4) decreases during postmortem aging in meat [49]. These findings highlight the importance of further investigation into the physiological and enzymatic processes driving metabolite changes during wet aging to better understand the mechanisms underlying the sweet aroma and meat flavor formation in Japanese Black cattle.

**Figure 7:**
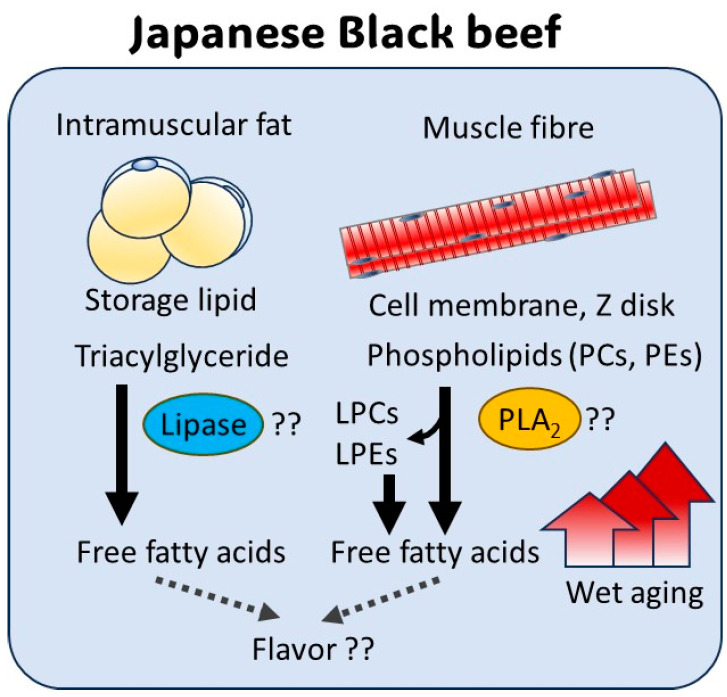
Pathway of free fatty acid (FFA) production during wet aging. Triacylglycerols (TGs) and phospholipids such as phosphatidylcholines (PCs) and phosphatidylethanolamines (PEs) undergo enzymatic degradation, primarily by lipase and phospholipase A2 (PLA_2_), resulting in FFA release and accumulation. The distinct lipid metabolism and enzymatic activity in intramuscular fat compared to muscle tissue suggest that FFA production pathways may vary between these tissues. Such differences may contribute to the observed between the longissimus thoracis and adductor muscle, as well as between Japanese Black and Holstein beef.

Various lipid-degrading enzymes are expressed in muscle and fat tissues, and specific lipases may contribute to FFA formation [42]. Since little is known about the physiological changes during postmortem aging or the structural alterations in membrane phospholipids, future research should focus on elucidating the role of FFAs in beef quality at meat processing centers [34] (Figure 7).

A key limitation of this study is the inherent heterogeneities in data from the metabolomics analysis of Japanese Black beef samples. These samples are derived from marbled tissues, which comprise a mixture of muscle fibers and intramuscular fat. The proportion of intramuscular fat varies between samples, resulting in higher CVs in metabolomics analysis compared to other breeds [10].

Future studies should address this variability by increasing the number of samples analyzed to achieve a more detailed understanding of the metabolites that contribute to beef flavor and quality. Furthermore, integrating advanced analytical techniques, such as spatial metabolomics and imaging technologies, could provide more precise insights into the distribution and interaction of metabolites within marbled tissues.

## 5. Conclusions

This study explored the effects of wet aging on Japanese Black and Holstein beef using metabolomics analysis. Wet aging significantly increased the concentration of flavor-related metabolites, including free amino acids and organic acids, in both breeds. FFAs, such as oleic and linoleic acids, showed marked increases, especially in Japanese Black beef, contributing to enhanced flavor characteristics. Additionally, the degradation of phospholipids, particularly LPC and LPE, highlighted the breakdown of cell membrane lipids during the aging process. These results suggest that the flavor development in Japanese Black and Holstein beef during wet aging is closely linked to the release of FFAs and other flavor-related metabolites. This study provides valuable insights for optimizing aging processes to improve meat quality in different cattle breeds.

## Data Availability

The data presented in this study are available on request from the corresponding author. The data are not publicly available due to patent issues.

## Supplementary Materials

The following supporting information can be downloaded at https://www.mdpi.com/article/10.3390/metabo15020094/s1, Figure S1: Photographs show Japanese Black and Holstein beef. Figure S2: Comprehensive analysis of phospholipid molecules during wet aging. Figure S3: Quantitative analysis of changes in free amino acids during wet aging. Figure S4: Quantitative analysis of nucleic acid-related metabolites during wet aging. Figure S5: Heat map analysis of metabolomics data. Table S1: Metabolite changes during wet aging of Japanese Black beef. Table S2: Metabolite changes during wet aging of Holstein beef. Table S3: Various changes in metabolites depend on cold storage time. Table S4: Quantitative analysis of free fatty acids of Holstein beef during wet aging. Reference [50] is cited in the Supplementary Material.

## Abbreviations

FFAs	Free fatty acids
GC–MS	Gas chromatography–mass spectrometry
LC–MS	Liquid chromatography–mass spectrometry
SD	Standard deviation
SE	Standard error
NS	Not significant
CV	Coefficient of variation
TG	Triacylglyceride
FA	Fatty acid
SFA	Saturated fatty acid
MUFA	Monounsaturated fatty acids
PUFA	Polyunsaturated fatty acids
PCs	Phosphatidylcholines
LPCs	Lysophosphatidylcholines
PEs	Phosphatidylethanolamines
LPEs	Lysophosphatidylethanolamines
PLA	Phospholipase A
