# Supplementary material for: Metabolites and Free Fatty Acids in Japanese Black Beef During Wet Aging
Source: Metabolites. 2025 Feb 3;15(2):94. doi: 10.3390/metabo15020094 (PMC11857383; doi:10.3390/metabo15020094)
Supplement: Supplementary file 1 [file metabolites-15-00094-s001.zip › metabolites-3343579-supplementary.pdf]

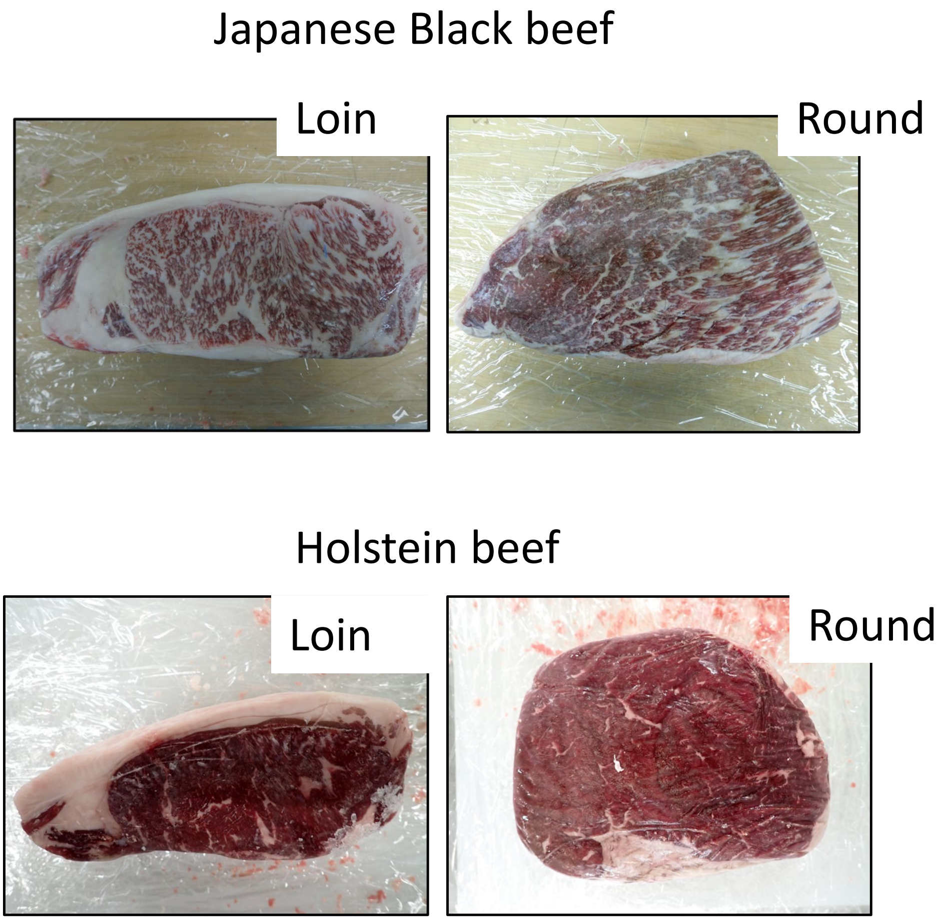

Figure S1. Photographs of Japanese Black and Holstein beef showing the longissimus thoracis (Loin) and adductor muscle (Round).

Japanese Black Loin

metabolites	Wet aging days 0	10	20	30	40
Fructose	31.16 ± 2.04	32.67 ± 1.68	33.83 ± 1.27	36.06 ± 1.91	37.18 ± 0.71
Glucose	3.66 ± 0.25	4.35 ± 0.34	4.56 ± 0.18	5.66 ± 0.41	4.72 ± 0.22
Lactic acid	241.37 ± 12.56	94.45 ± 22.06	37.11 ± 0.64	38.65 ± 1.52	170.3 ± 20.29
Inosine	18.95 ± 2.03	5.7 ± 1.32	2.84 ± 0.19	1.35 ± 0.11	3.97 ± 0.44
Hypoxanthine	27.32 ± 2.03	19.65 ± 2.84	17.15 ± 0.53	22.02 ± 0.49	49.63 ± 3.79
Xanthine	0.33 ± 0.08	0.9 ± 0.34	0.4 ± 0.27	0.49 ± 0.32	0.54 ± 0.13
Fructose 6-phosphate	1.13 ± 0.44	19.08 ± 2.47	20.48 ± 1.08	14.68 ± 1.09	3.82 ± 1.3
Ribulose 1,5-diphosphate	0.06 ± 0.02	0.5 ± 0.06	1.23 ± 0.15	2.24 ± 0.1	0.55 ± 0.13
Ribulose-5-phosphate	0.05 ± 0.01	0.85 ± 0.11	1 ± 0.03	0.43 ± 0.04	0.15 ± 0.04
Ornithine	0.48 ± 0.05	2.03 ± 0.42	3.36 ± 0.4	3.19 ± 0.28	1.68 ± 0.28
Oxalic acid	0.53 ± 0.02	0.32 ± 0.05	0.22 ± 0.02	0.19 ± 0.02	0.58 ± 0.05
Succinic acid	34.12 ± 1.59	37.78 ± 2.33	39.62 ± 0.67	40.57 ± 1.55	34.59 ± 2.56
Citric acid	0.14 ± 0.0	0.23 ± 0.02	0.5 ± 0.04	0.68 ± 0.06	0.23 ± 0.03
Glycerol	146 ± 7.95	65.33 ± 7.84	30.75 ± 3.45	40.55 ± 1.58	119.06 ± 9.66
Glyceric acid	0.87 ± 0.05	1.63 ± 0.16	2.35 ± 0.07	3.74 ± 0.14	2.33 ± 0.18

Japanese Black Round

metabolites	Wet aging days 0	10	20	30	40
Fructose	35.32 ± 4.48	46.23 ± 2.61	50.48 ± 3.09	33.06 ± 1.75	48.42 ± 2.86
Glucose	4.71 ± 0.56	6.11 ± 0.41	6.52 ± 0.41	4.23 ± 0.2	5.64 ± 0.32
Lactic acid	111.68 ± 15.64	163.13 ± 17.36	154.73 ± 15.01	105.58 ± 9.18	104.36 ± 6.58
Inosine	16.53 ± 2.56	13.01 ± 0.98	10.5 ± 0.81	5.37 ± 0.34	10.58 ± 0.89
Hypoxanthine	26.36 ± 4.08	42.75 ± 3.47	48.49 ± 3.88	35.57 ± 2.63	35.78 ± 4.5
Xanthine	0.27 ± 0.05	0.29 ± 0.06	0.18 ± 0.04	0.06 ± 0.01	0.34 ± 0.09
Fructose 6-phosphate	7.64 ± 0.73	2.08 ± 0.49	1.47 ± 0.54	2.5 ± 0.57	1.42 ± 0.46
Ribulose 1,5-diphosphate	0.12 ± 0.01	0.05 ± 0.01	0.05 ± 0.01	0.2 ± 0.05	0.18 ± 0.06
Ribulose-5-phosphate	0.33 ± 0.05	0.16 ± 0.04	0.13 ± 0.04	0.15 ± 0.03	0.07 ± 0.01
Ornithine	2.45 ± 0.37	0.59 ± 0.05	0.55 ± 0.06	1.13 ± 0.14	0.98 ± 0.08
Oxalic acid	0.36 ± 0.03	0.42 ± 0.03	0.45 ± 0.03	0.4 ± 0.03	0.41 ± 0.02
Succinic acid	15.95 ± 0.27	14.65 ± 1.0	14.97 ± 0.73	17.27 ± 0.66	21 ± 0.76
Citric acid	0.11 ± 0.01	0.09 ± 0.01	0.08 ± 0.0	0.13 ± 0.02	0.12 ± 0.01
Glycerol	69.37 ± 7.52	75.25 ± 11.58	101.68 ± 6.84	82.12 ± 6.42	73.95 ± 4.06
Glyceric acid	0.53 ± 0.05	0.54 ± 0.04	0.63 ± 0.03	0.6 ± 0.03	3.37 ± 0.23

Table S1. Metabolite changes during wet aging of Japanese Black beef. Error bars represent the mean ± SE.

Holstein Loin

metabolites	Wet aging days				
	0	10	20	30	40
Fructose	3.49 ± 0.09	5.2 ± 0.4	9.71 ± 0.3	11.18 ± 0.97	10.87 ± 0.54
Glucose	3.42 ± 0.09	3.45 ± 0.14	5.62 ± 0.17	6.56 ± 0.32	7.66 ± 0.37
Lactic acid	49.77 ± 1.25	42.81 ± 0.6	46.56 ± 2.25	42.45 ± 1.0	48.15 ± 1.69
Inosine	3.41 ± 0.09	3.1 ± 0.06	4.26 ± 0.22	4.24 ± 0.06	4.5 ± 0.17
Hypoxanthine	3.32 ± 0.15	5.6 ± 0.14	9.09 ± 0.41	11.33 ± 0.33	15.65 ± 0.53
Xanthine	0.09 ± 0.0	0.14 ± 0.0	0.19 ± 0.01	0.24 ± 0.01	0.35 ± 0.02
Fructose 6-phosphate	8.95 ± 0.32	7.16 ± 0.53	9.11 ± 0.18	7.87 ± 0.44	6.04 ± 0.33
Ribulose 1,5-diphosphate	0.08 ± 0.0	0.21 ± 0.02	0.29 ± 0.01	0.34 ± 0.02	0.41 ± 0.03
Ribulose-5-phosphate	0.19 ± 0.01	0.2 ± 0.01	0.21 ± 0.01	0.19 ± 0.01	0.2 ± 0.01
Ornithine	0.67 ± 0.05	0.6 ± 0.03	0.65 ± 0.04	0.66 ± 0.02	1.47 ± 0.07
Oxalic acid	3.69 ± 0.07	3.52 ± 0.2	4.28 ± 0.39	4.13 ± 0.15	4.7 ± 0.14
Succinic acid	15.35 ± 1.08	11.93 ± 0.66	15.27 ± 0.91	14.78 ± 0.95	13.55 ± 1.74
Citric acid	0.11 ± 0.01	0.05 ± 0.01	0.06 ± 0.01	0.11 ± 0.01	0.2 ± 0.02
Glycerol	35.02 ± 1.27	26.93 ± 0.73	34.33 ± 1.52	37.94 ± 0.65	40.32 ± 1.09
Glyceric acid	0.33 ± 0.02	0.68 ± 0.11	1.53 ± 0.14	1.25 ± 0.11	1.27 ± 0.12

Holstein Round

metabolites	Wet aging days				
	0	10	20	30	40
Fructose	3.21 ± 0.13	5.42 ± 0.22	6.57 ± 0.34	7.4 ± 0.53	8.32 ± 0.73
Glucose	3.02 ± 0.12	3.79 ± 0.11	4.34 ± 0.16	5.07 ± 0.25	5.66 ± 0.38
Lactic acid	18.92 ± 0.4	19.72 ± 0.45	20.54 ± 0.59	25.55 ± 0.84	24.21 ± 0.36
Inosine	3.7 ± 0.09	3.69 ± 0.05	4.29 ± 0.3	4.46 ± 0.09	4.33 ± 0.11
Hypoxanthine	2.36 ± 0.07	6.53 ± 0.26	8.57 ± 0.42	11.82 ± 0.26	13.69 ± 0.21
Xanthine	0.06 ± 0.0	0.1 ± 0.01	0.17 ± 0.02	0.15 ± 0.01	0.2 ± 0.01
Fructose 6-phosphate	11.02 ± 0.56	6.73 ± 0.1	4.89 ± 0.2	4.14 ± 0.23	3.95 ± 0.25
Ribulose 1,5-diphosphate	0.05 ± 0.0	0.11 ± 0.01	0.12 ± 0.0	0.17 ± 0.01	0.22 ± 0.01
Ribulose-5-phosphate	0.23 ± 0.02	0.29 ± 0.02	0.26 ± 0.02	0.29 ± 0.02	0.28 ± 0.01
Ornithine	0.89 ± 0.02	1.12 ± 0.05	1.03 ± 0.09	0.97 ± 0.03	1.19 ± 0.05
Oxalic acid	2.43 ± 0.04	2.57 ± 0.05	2.81 ± 0.14	3.94 ± 0.13	4.58 ± 0.14
Succinic acid	8.81 ± 0.54	8.7 ± 1.28	11.53 ± 0.68	14.24 ± 1.04	13.89 ± 0.65
Citric acid	0.06 ± 0.02	0.05 ± 0.0	0.1 ± 0.0	0.16 ± 0.01	0.23 ± 0.03
Glycerol	19.02 ± 0.3	20.04 ± 0.4	20.97 ± 0.44	26.17 ± 0.88	24.81 ± 0.43
Glyceric acid	0.25 ± 0.0	0.24 ± 0.0	0.33 ± 0.02	0.41 ± 0.01	0.48 ± 0.01

Table S2. Metabolite changes during wet aging of Holstein beef. Error bars represent the mean ± SE.

Japanese Black beef (Loin)

Name (mg/100g)	Abbreviation	Day 0	Day 10	Day 20	Day 30	Day 40
Myristic acid	C14:0	6.0 ± 1.5	6.7 ± 1.3	7.1 ± 1.9	8.5 ± 1.7	9.4 ± 4.9
Myristoleic acid	C14:1	0.0 ± 0.0	0.0 ±0.0	0.5 ± 0.2	1.3 ± 0.4	1.3 ± 0.2
Pentadecanoic acid	C15:0	0.0 ± 0.0	0.0 ± 0.0	0.7 ± 0.2	1.3 ± 0.2	2.3 ± 0.6
Palmitic acid	C16:0	33.6 ± 2.2	38.4 ± 2.2	38.8 ± 2.9	45.2 ± 4.3	43.7 ± 5.0
Palmitoleic acid	C16:1	3.6 ± 0.9	5.5 ± 0.8	5.4 ± 1.3	6.9 ± 1.7	7.7 ± 2.9
Margaric acid	C17:0	0.0 ± 0.0	0.0 ± 0.0	0.0 ± 0.0	0.0 ± 0.0	0.9 ± 0.4
Stearic acid	C18:0	22.5 ± 2.1	21.6 ± 2.5	22.3 ± 1. 5	24.8 ± 3.7	20.9 ± 3.7
Oleic acid	C18:1	46.9 ± 6.0	62.1 ± 9.5	65.2 ± 7.6	73.8 ± 7.0	69.6 ± 8.5
Linoleic acid	C18:2	9.2 ± 1.5	10.4 ± 1.5	9.6 ± 2.8	9.5 ± 3.4	9.2 ± 2.9
Linolenic acid	C18:3	0.0 ± 0.0	0.0 ± 0.0	0.0 ± 0.0	0.0 ± 0.0	0.0 ± 0.0
Dihomo-γ-linolenic acid	C20：3	0.0 ± 0.0	0.0 ± 0.0	0.0 ± 0.0	0.0 ± 0.0	0.0 ± 0.0
Arachidonic acid	C20：4	0.0 ± 0.0	0.0 ± 0.0	0.0 ± 0.0	1.3 ± 0.0	2.8 ± 0.6

Japanese Black beef (Round)

Name (mg/100g)	Abbreviation	Day 0	Day 10	Day 20	Day 30	Day 40
Myristic acid	C14:0	2.3 ± 0.7	4.2 ± 0.9	3.6 ± 2.2	5.3 ± 1.0	3.8 ± 0.7
Myristoleic acid	C14:1	0.0 ± 0.0	0.0 ± 0.0	0.0 ± 0.0	0.0 ± 0.0	0.0 ± 0.0
Pentadecanoic acid	C15:0	0.0 ± 0.0	0.0 ± 0.0	0.0 ± 0.0	0.0 ± 0.0	0.0 ± 0.0
Palmitic acid	C16:0	18.5 ± 0.9	21.1 ± 1.7	23.9 ± 3.8	31.3 ± 2.4	32.1 ± 2.1
Palmitoleic acid	C16:1	2.3 ± 0.2	2.7 ± 0.7	3.8 ± 1.7	5.8 ± 2.4	4.3 ± 1.0
Margaric acid	C17:0	0.0 ± 0.0	0.0 ± 0.0	0.0 ± 0.0	0.0 ± 0.0	0.0 ± 0.0
Stearic acid	C18:0	9.5 ± 0.9	11.8 ± 1.6	12.5 ± 2.1	15.4 ± 2.8	14.7 ± 1.4
Oleic acid	C18:1	27.6 ± 3.6	32.7 ± 2.4	34.8 ± 2.8	46.9 ± 3.2	45.5 ± 6.8
Linoleic acid	C18:2	8.4 ± 2.9	8.3 ± 3.4	10.8 ± 2.6	11.9 ± 2.7	13.7 ± 5.1
Linolenic acid	C18:3	0.0 ± 0.0	0.0 ± 0.0	0.0 ± 0.0	0.0 ± 0.0	0.0 ± 0.0
Dihomo-γ-linolenic acid	C20：3	0.0 ± 0.0	0.0 ± 0.0	0.0 ± 0.0	0.0 ± 0.0	0.0 ± 0.0
Arachidonic acid	C20：4	3.2 ± 0.0	0.0 ± 0.0	0.0 ± 0.0	3.0 ± 0.0	7.7 ± 2.3

Table S3. Quantitative analysis of free fatty acids of Japanese Black beef during wet aging. Free fatty acids extracted from longissimus thoracis (Loin) and adductor muscle (Round) were analyzed using gas chromatography. The table values represent the mean ± standard deviation (n = 5).

Holstein beef (Loin)

Name (mg/100g)	Abbreviation	Day 0	Day 10	Day 20	Day 30	Day 40
Myristic acid	C14:0	0.8 ± 0.0	1.3 ± 0.0	1.5 ± 0.0	1.4 ± 0.0	1.7 ± 0.0
Myristoleic acid	C14:1	0.0 ± 0.0	0.0 ± 0.0	0.0 ± 0.0	0.0 ± 0.0	0.2 ± 0.0
Pentadecanoic acid	C15:0	0.0 ± 0.0	0.0 ± 0.0	0.0 ± 0.0	0.0 ± 0.0	0.0 ± 0.0
Palmitic acid	C16:0	7.0 ± 0.6	13.7 ± 0.7	16.1 ± 1.2	12.5 ± 0.5	16.4 ± 0.6
Palmitoleic acid	C16:1	0.5 ± 0.3	0.1 ± 0.1	0.5 ± 0.2	1.6 ± 0.2	2.2 ± 0.3
Margaric acid	C17:0	0.0 ± 0.0	0.0 ± 0.0	0.0 ± 0.0	0.0 ± 0.0	0.0 ± 0.4
Stearic acid	C18:0	4.0 ± 0.4	9.5 ± 1.3	9.3 ± 0.7	7.7 ± 0.6	8.5 ± 1.3
Oleic acid	C18:1	4.5 ± 0.7	4.9 ± 1.0	6.4 ± 2.6	15.0 ± 0.7	20.3 ± 1.7
Linoleic acid	C18:2	0.9 ± 0.3	1.7 ± 1.0	1.7 ± 0.7	4.7 ± 1.2	5.4 ±1.2
Linolenic acid	C18:3	0.0 ± 0.0	0.0 ± 0.0	0.0 ±0.0	0.0 ± 0.0	0.0 ±0.0
Dihomo-γ-linolenic acid	C20：3	0.0 ± 0.0	0.0 ±0.0	0.0 ± 0.0	0.0 ± 0.0	0.0 ±0.0
Arachidonic acid	C20：4	0.0 ± 0.0	0.0 ± 0.0	0.0 ± 0.0	0.0 ± 0.0	0.0 ± 2.1

Holstein beef (Round)

Name (mg/100g)	Abbreviation	Day 0	Day 10	Day 20	Day 30	Day 40
Myristic acid	C14:0	0.7 ± 0.0	1.3 ± 0.0	1.3 ± 0.0	0.8 ± 0.0	1.0 ± 0.0
Myristoleic acid	C14:1	0.0 ± 0.0	0.2 ± 0.0	0.2 ± 0.0	0.3 ± 0.0	0.3 ± 0.0
Pentadecanoic acid	C15:0	0.1 ± 0.0	0.4 ± 0.0	0.7 ± 0.0	0.7 ± 0.0	0.8 ± 0.0
Palmitic acid	C16:0	9.5 ± 1.8	10.5 ± 3.3	17.6 ± 2.3	19.6 ± 1.9	24.5 ± 2.9
Palmitoleic acid	C16:1	0.3 ± 0.1	0.7 ± 0.6	1.7 ± 0.5	1.2 ± 0.1	1.3 ± 0.4
Margaric acid	C17:0	0.0 ± 0.0	0.4 ± 0.0	0.5 ± 0.0	0.4 ± 0.0	0.6 ± 0.0
Stearic acid	C18:0	5.3 ± 0.4	5.8 ± 1.3	7.8 ± 0.7	9.5 ± 0.6	12.6 ± 1.3
Oleic acid	C18:1	4.6± 2.6	7.8 ± 3.0	17.4 ± 1.4	18.0 ± 3.2	20.5 ± 2.3
Linoleic acid	C18:2	1.9 ± 1.2	2.0 ± 1.6	7.9 ± 0.6	14.5 ± 4.2	14.3 ± 3.1
Linolenic acid	C18:3	0.0 ± 0.0	0.0 ± 0.0	0.0 ± 0.0	0.0 ± 0.0	0.0 ± 0.0
Dihomo-γ-linolenic acid	C20：3	0.0 ± 0.0	0.0 ± 0.0	1.2 ± 0.0	2.0 ± 0.0	1.9 ± 0.0
Arachidonic acid	C20：4	0.0 ± 0.0	0.5 ± 0.0	2.6 ± 0.0	4.4 ± 0.0	4.0 ± 2.0

Table S4. Quantitative analysis of free fatty acids of Holstein beef during wet aging. Free fatty acids extracted from longissimus thoracis (Loin) and adductor muscle (Round) were analyzed using gas chromatography. The table values represent the mean ± standard deviation (n = 5).

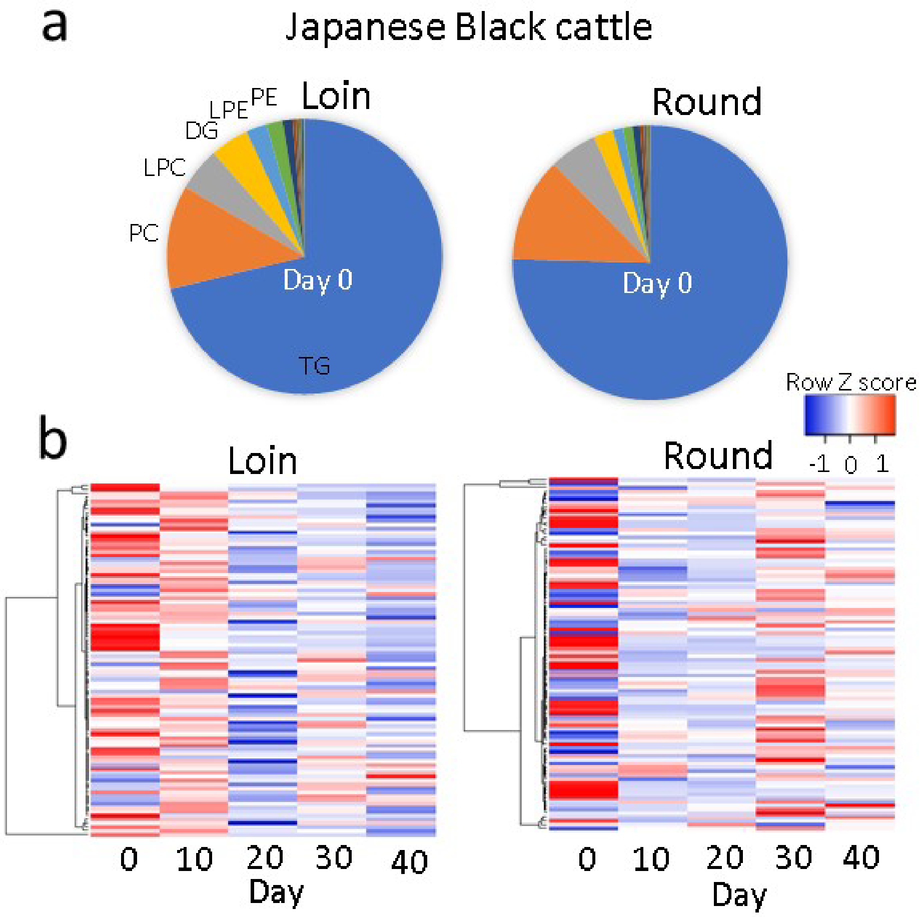

Figure S2. Comprehensive analysis of phospholipids molecules during wet aging. (**a**) Lipid Composition of the longissimus thoracis (Loin) and adductor muscle (Round) in Japanese Black cattle, detected by liquid chromatography-mass spectrometry. The pie chart shows the molecular species of each lipid type on Day 0. For the Loin, the coefficient of variation was as follows: total PC (12.6%), total LPC (21.1%), total PE (21.8%), and total LPE (18.0%). For the Round, the CV values were total PC (14.1%), total LPC (23.6%), total PE (15.5%), and total LPE (19.7%). (**b**) Heatmap of lipid metabolites in Japanese Black cattle. Blue indicates low concentrations, and red indicates high concentrations of lipid molecules. Color gradients reflect changes in metabolite concentrations over the wet aging period.

Abbreviations: triacylglyceride (TG), phosphatidylcholine (PC), lysophosphatidylcholine (LPC), diacylglyceride (DG), lysophosphatidylethanolamine (LPE), phosphatidylethanolamine (PE). Heatmaps were drawn using Heatmapper [50]* (http://www.heatmapper.ca/, accessed on 1 December 2023).

* 50. Babicki, S.; Arndt, D.; Marcu, A.; Liang, Y.; Grant, J.R.; Maciejewski, A.; Wishart, D.S. Heatmapper: web-enabled heat mapping for all. *Nucleic Acids Res.* **2016**, *44*, W147–153, doi:10.1093/nar/gkw419.

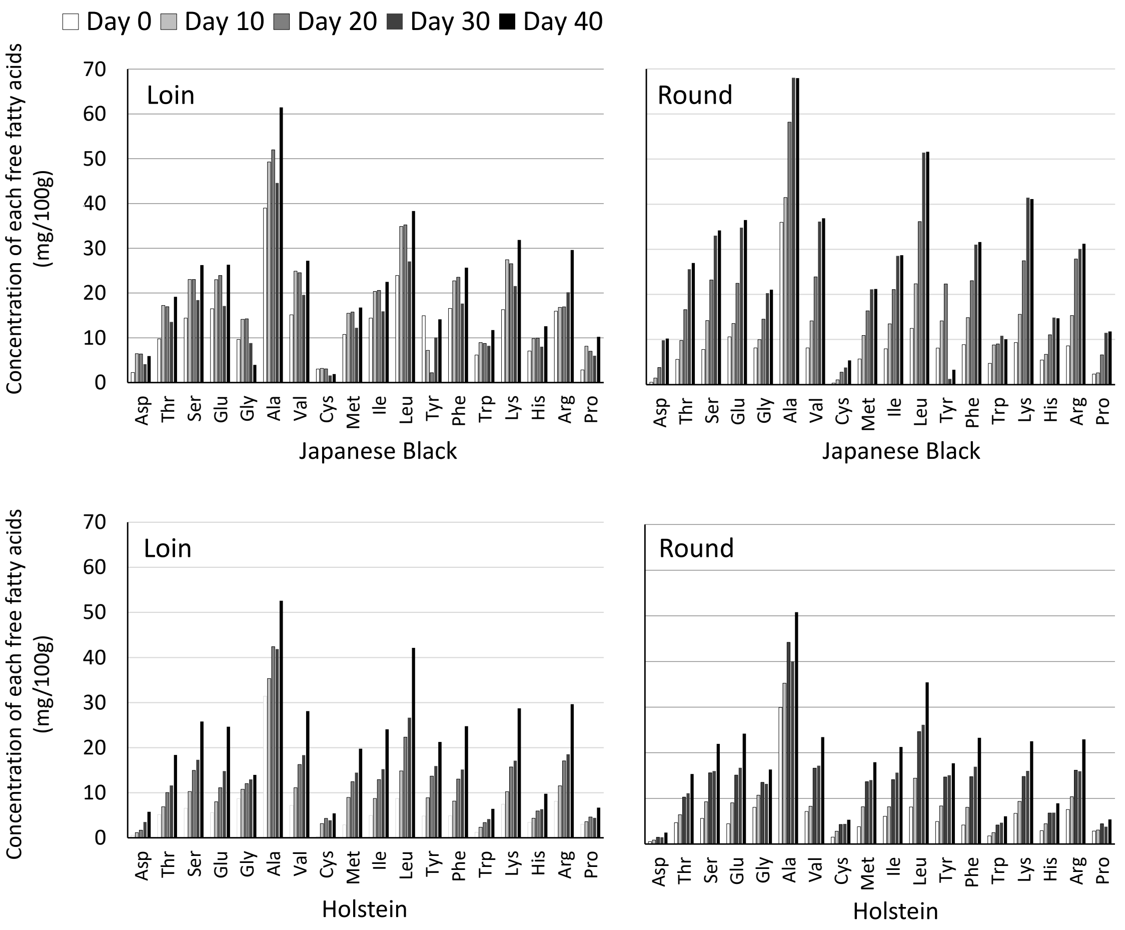

Figure S3. Quantitative analysis of changes in free amino acids during wet aging. Free amino acids extracted from longissimus thoracis (Loin) and adductor muscle (Round) were analyzed by gas chromatography. The values in the graph represent the mean of each metabolite (n = 5).

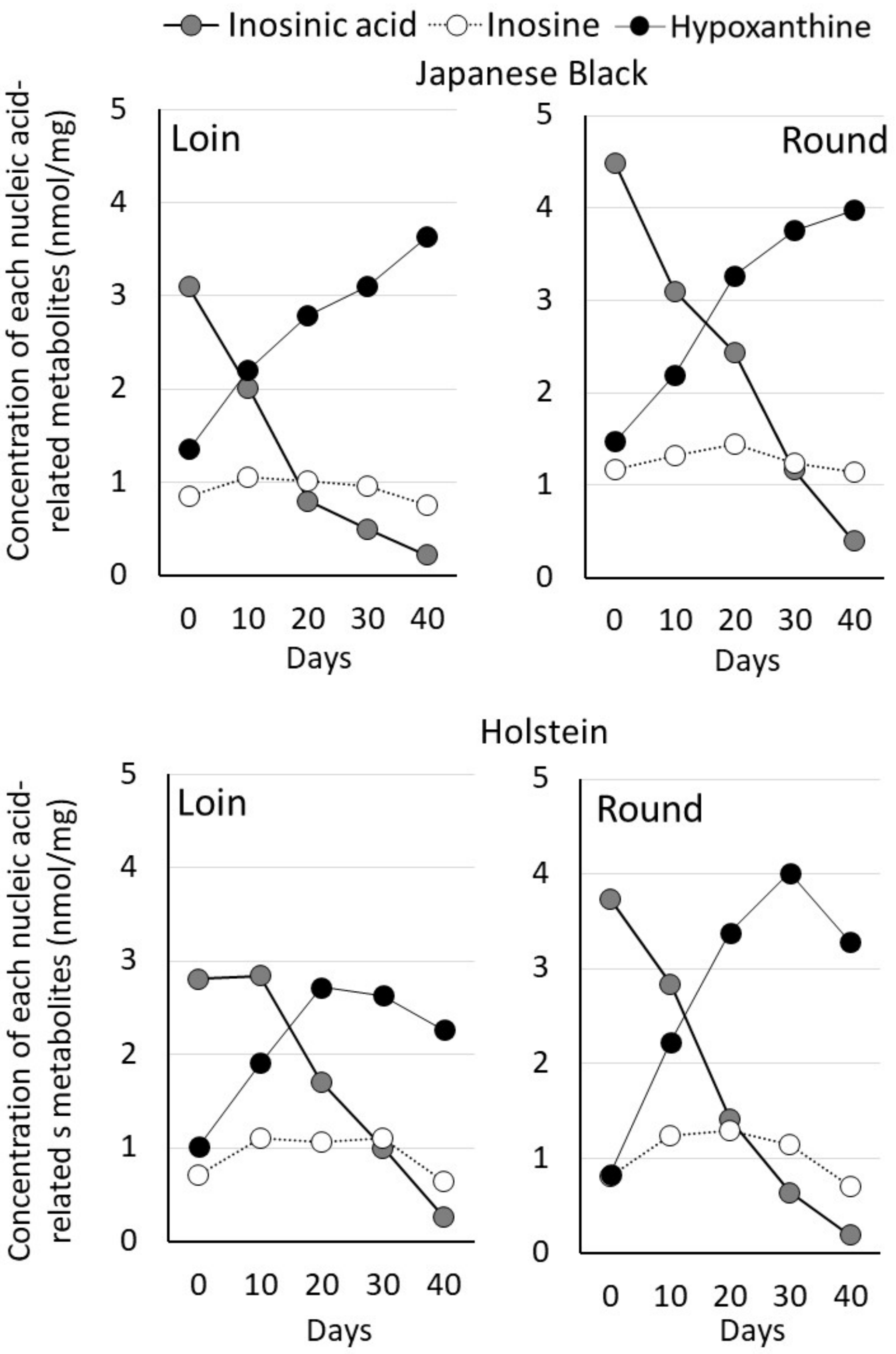

Figure S4. Quantitative analysis of nucleotide-related metabolites during wet aging. Nucleic acid-related metabolites in the longissimus thoracis (Loin) and adductor muscle (Round) were analyzed using high-performance liquid chromatography. The values in the graph represent the mean of each metabolite (n = 5).

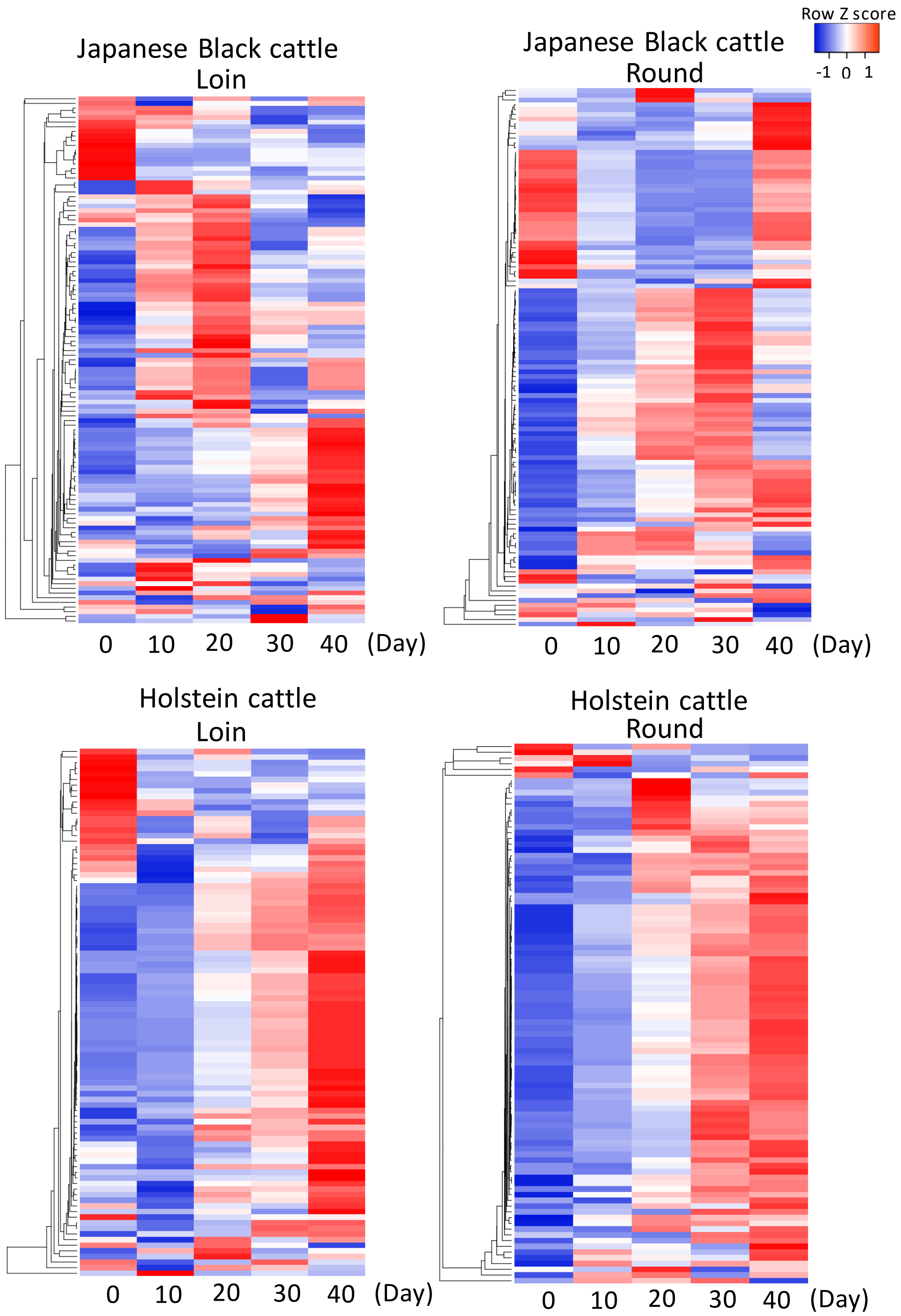

Figure S5. Heatmap analysis of metabolomics data. Heatmap analysis visualizes increases and decreases in metabolite data obtained through gas chromatography-mass spectrometry. Heatmaps were drawn using Heatmapper* (http://www.heatmapper.ca/, accessed on 1 December 2023).
